# Association of continuity of care with treatment adherence and quality of life in patients with chronic kidney disease and hyperkalemia: A retrospective study

**DOI:** 10.1097/MD.0000000000049514

**Published:** 2026-07-03

**Authors:** Yu Yan, Juan Liu, Guifang Liu

**Affiliations:** a Blood Purification Center, The First Affiliated Hospital of University of South China, Hengyang City, Hunan Province, China; b Ward 2, Department of Nephrology, The Second Affiliated Hospital of the University of South China, Hengyang City, Hunan Province, China.

**Keywords:** chronic kidney disease, compliance, continuity of care, hyperkalemia

## Abstract

This retrospective study evaluated whether a continuity-of-care nursing model was associated with improved adherence and quality of life in patients with chronic kidney disease complicated by hyperkalemia. Medical records of patients hospitalized between January 2021 and December 2023 were reviewed. Based on documented discharge planning and postdischarge follow-up intensity, patients were categorized into a control group receiving routine education and standard follow-up, and a experimental group receiving enhanced transitional and postdischarge nursing. The continuity-of-care approach incorporated structured guidance on fluid restriction, dietary potassium and sodium control, self-care training, disease management, medication counseling, lifestyle modification, weight management, and scheduled follow-up. Outcomes extracted from follow-up documentation included multidomain adherence (fluid restriction, potassium intake control, self-care behaviors, disease management, and sodium intake), preventive-treatment adherence (medication adherence, lifestyle modification, weight management, and follow-up attendance), psychological status assessed by the self-rating anxiety scale and self-rating depression scale, overall quality of life, and satisfaction with care. Compared with usual care, the continuity-of-care group demonstrated higher adherence across multiple domains and better preventive-treatment compliance (all *P* < .001). After the care period, self-rating anxiety scale and self-rating depression scale scores were lower in the continuity-of-care group, indicating reduced anxiety and depressive symptoms. Quality of life scores and satisfaction with nursing care were higher in the continuity-of-care group than in the usual-care group (*P* < .05). These findings suggest that continuity of care is associated with improved adherence and patient-reported outcomes in chronic kidney disease patients with hyperkalemia, although prospective studies are needed to confirm causality.

## 1. Introduction

Chronic kidney disease (CKD) represents a burgeoning public health challenge on a global scale, with the global burden of disease study indicating its prevalence among approximately 10% to 15% of the adult population worldwide, a figure that continues to ascend.^[1]^ The trajectory of CKD frequently culminates in a spectrum of complications, with hyperkalemia – an eminent electrolyte derangement – emerging as a prevalent issue among afflicted patients.^[2]^ Hyperkalemia not only escalates the susceptibility to cardiovascular events like arrhythmias but also exerts a profound impact on patients’ quality of life and daily functionality. The management of complications and the enhancement of the quality of life for CKD patients have posed formidable challenges to the healthcare system in light of the escalating patient numbers.^[3]^

In recent times, the paradigm of continuity of care has emerged as an innovative nursing model garnering considerable attention in the realm of chronic patient management. At its core, continuity-of-care endeavors to furnish uninterrupted and comprehensive nursing services postdischarge to foster patients’ self-management capabilities and instigate health behavior modifications.^[4,5]^ The continuum of care model encompasses tailored health education predischarge, routine domiciliary visits, remote support facilitated through communication platforms such as telephone and WeChat, and the establishment of a closely-knit collaborative network spanning hospitals, communities, and families.^[6,7]^ The primary objective of this model is to facilitate patients’ adherence to medical directives and enhance their lifestyle through a continuum of care and support, thereby augmenting treatment efficacy and quality of life.^[8,9]^ An observational study utilizing Norwegian registry data investigated the relationship between continuity of care and mortality among patients with asthma, chronic obstructive pulmonary disease, diabetes, and heart failure. The results indicated that higher continuity of care was associated with lower mortality rates, which also provides a reference for the intervention methods of this study.^[10,11]^

Nevertheless, the empirical evidence regarding the efficacy of continuity of care in patients grappling with CKD and hyperkalemia remains scant. Hence, this study endeavors to elucidate the impact of continuity of care on this specific patient cohort, with the aim of furnishing more scientifically grounded and efficacious strategies for clinical nursing practice, thereby ameliorating the overall health status and quality of life of patients.

## 2. Data and methods

### 2.1. Research subjects

This study was approved by the Ethics Committee of The Second Affiliated Hospital of Hengyang Medical School. Because this was a retrospective study using existing medical records, the requirement for written informed consent was waived by the Institutional Review Board and Ethics Committee. All patient information was anonymized before analysis, and the data were handled confidentially in accordance with institutional ethical requirements and the principles of the Declaration of Helsinki. No personally identifiable information was used in the analysis or presented in this manuscript. A retrospective cohort of 120 patients with a documented diagnosis of chronic kidney disease and hyperkalemia who were admitted to the hospital between January 2021 and December 2023 was identified from electronic medical records. Patients were categorized into a usual-care (control) group and a continuity-of-care (comparison) group according to the type and intensity of nursing management and follow-up documented during hospitalization and after discharge, rather than by random allocation. Ethical approval was obtained from the ethical committee of our university hospital, and permission was also obtained from the university hospital where the study was carried out. The requirement for informed consent was waived by the Institutional Review Board and Ethics Committee.

Inclusion criteria:

① Diagnosed with chronic kidney disease stages 3 to 5.^[11]^
② Newly hospitalized with a blood potassium level of ≥5.0 mmol/L.^[12]^
③ Age ≥ 18 years.

Exclusion criteria:

① Individuals undergoing hemodialysis or peritoneal dialysis.
② Those with unclear language expression or communication impediments.
③ Individuals with mental disorders or cognitive impairments.
④ Patients with comorbidities such as tumors, tuberculosis, severe trauma, etc.

Drop out criteria:

① Individuals whose condition significantly altered during the study, no longer meeting inclusion criteria.
② Patients who succumbed or voluntarily requested withdrawal.
③ Individuals lost to follow-up for various reasons.

### 2.2. Research methods

Both the control and experimental groups underwent a 3-month intervention. The control group received standard discharge education and follow-up, whereas the experimental group was subjected to a continuity-of-care model.

Control group: Patients received conventional nursing care encompassing health education on medication, dietary restrictions, daily regimens, self-monitoring of vital signs, and rehabilitative training predischarge. Patients were advised to seek medical attention if any discomfort arose. Telephone follow-ups were conducted at 1 and 3 months postdischarge.

Experimental group: Building upon the control group’s framework, the continuity-of-care model was augmented with the following specific interventions^[10,11]^:

① Predischarge nursing: Tailored health education on chronic kidney disease and hyperkalemia was dispensed 1 to 2 days antecedent to discharge. The medical team proffered individualized elucidations to patients, considering their unique circumstances and the guidelines pertaining to hyperkalemia in chronic kidney disease. The educational content encompassed risk factors, perils, medication management, physical activity, and preventive healthcare knowledge concerning hyperkalemia.
② Regular follow-up and guidance: Biweekly home visits were conducted to monitor patient progress. Subsequently, personalized health education and self-management skills guidance were dispensed based on individual patient circumstances through WeChat public accounts and groups. This approach heightened the frequency of patient–provider communication and delineated rehabilitation objectives.
③ Formulation and evaluation of continuity-of-care intervention plan: In instances where patients exhibited unfavorable lifestyle alterations and cultivated healthy behaviors, the medical team aided in setting health objectives and crafting personalized health behavior nursing strategies commensurate with patients’ actual situations. Patients who diligently implemented the plans were encouraged, with self-monitoring, management, and evaluation undertaken.
④ Family-community-hospital support system: Leveraging the patient’s family, community milieu, and hospital infrastructure, this support system delivered consistent and tailored nursing care across diverse settings. The social environment of patients grappling with chronic kidney disease and hyperkalemia was assessed, with health education dispensed to patients and their families. Patients and their peers were encouraged to foster a communal environment, providing emotional and informational support to one another, while monitoring and evaluating changes in patients’ health behaviors.

### 2.3. Observational indices

① Renal disease diet compliance: The renal adherence behavior questionnaire was employed to gauge adherence to the renal disease diet.^[13]^ Comprising 5 dimensions and 25 items, the questionnaire was scored using the Likert 5-point method, ranging from 1 (“Never”) to 5 (“Always”). The Cronbach α coefficients for each dimension ranged from 0.56 to 0.80.
② Preventive treatment compliance: This metric delineates patients’ adherence to medical directives concerning the accurate timing and dosage of medication and other medical instructions. The compliance spectrum encompasses medication adherence, lifestyle enhancement, weight management, and regular follow-up visits, totaling 14 items and 12 points. A higher score signifies enhanced compliance. Medication adherence is appraised on a 4-point scale, lifestyle improvement compliance on a 4-point scale, weight control compliance on a 2-point scale, and regular follow-up compliance on a 2-point scale, with specifics detailed in Table 1. The questionnaire underwent evaluation by experts from the Department of Nephrology and Statistics, affirming its content validity and test-retest reliability.

**Table 1:** Scoring of treatment compliance assessment.

Item	4	3	2	1	0
Medication	Regular medication every week	Missed medication once a week	Missed medication twice a week	Missed medication 3 times or more per week	Stopped medication on their own
Lifestyle	No smoking or quit smoking for over half a year, no alcohol or quit drinking for over half a year, reasonable diet, exercise 3–5 times a week	Meets three of the above	Meets two of the above	Meets one of the above	No improvement
Weight control			BMI < 24 kg/m^2^	BMI ≥ 24 kg/m^2^	
Regular follow-up			Follow-up as required	Did not follow up as required	

In the study, the questionnaire’s content validity was determined to be 0.86, and the test–retest reliability Kappa coefficient was found to be 0.83, as assessed by a panel of 3 nursing experts from the Department of Nephrology and 1 expert from the Statistics Department.

③ Negative emotions: Regarding negative emotions, anxiety and depression were evaluated using the self-rating anxiety scale (SAS) and the self-rating depression scale (SDS), both comprising 20 items with a total score of 100 points each. The threshold values for anxiety and depression were set at 53 and 50 points, respectively, with higher scores indicating more pronounced negative emotional states.
④ Quality of life and nursing satisfaction: Assessed using the short form 36-item health survey (SF-36) and the nursing satisfaction questionnaire.^[14]^ The 36-item short form health survey comprises 8 dimensions and 36 items, with scores ranging from 0 to 100, where higher scores correspond to a better quality of life. The Nursing Satisfaction Questionnaire, on the other hand, evaluates patients’ satisfaction with hospital services, with scores categorized as satisfied (>80 points), fairly satisfied (60–80 points), or dissatisfied (<60 points).

### 2.4. Statistical methods

SPSS 25.0 software (IBM Corp.) was used for statistical analysis. The normality of continuous variables was assessed using the Shapiro–Wilk test and visual inspection of histograms and Q–Q plots. Homogeneity of variance was evaluated using Levene test. Normally distributed continuous variables were expressed as mean ± standard deviation and compared using independent-sample *t* tests. For comparisons before and after the intervention within the same group, paired-sample *t* tests were used when appropriate. Non-normally distributed continuous variables were expressed as medians and interquartile ranges and analyzed using nonparametric tests. Categorical variables were presented as numbers and percentages and compared using chi-square tests or Fisher exact tests, as appropriate. A 2-sided *P* value < .05 was considered statistically significant.

## 3. Results

### 3.1. Research subjects

Notably, the general characteristics of both cohorts, encompassing age, gender, duration of illness, and disease staging, exhibited no significant disparities and were deemed comparable (*P* > .05), as delineated in Table 2.

**Table 2:** Comparison of general information between 2 groups before intervention.

Category		Control group (n = 60)	Experimental group (n = 60)	*P*
Gender	Male	32	35	.581
	Female	28	25	
Age		40.43 ± 5.00	41.62 ± 6.21	.252
Educational level	Below primary school	14	19	.493
	Junior/senior high school	32	26	
	College and above	14	15	
Marital status	With spouse	45	48	.512
	Without spouse	15	12	
Serum potassium (mmol/L)		6.20 ± 0.78	6.28 ± 0.82	.592
Disease staging, n (%)	Stage 3	22	19	
	Stage 4	25	29	.757
	Stage 5	13	12	

### 3.2. Renal disease patients’ dietary compliance

**Figure 1.**
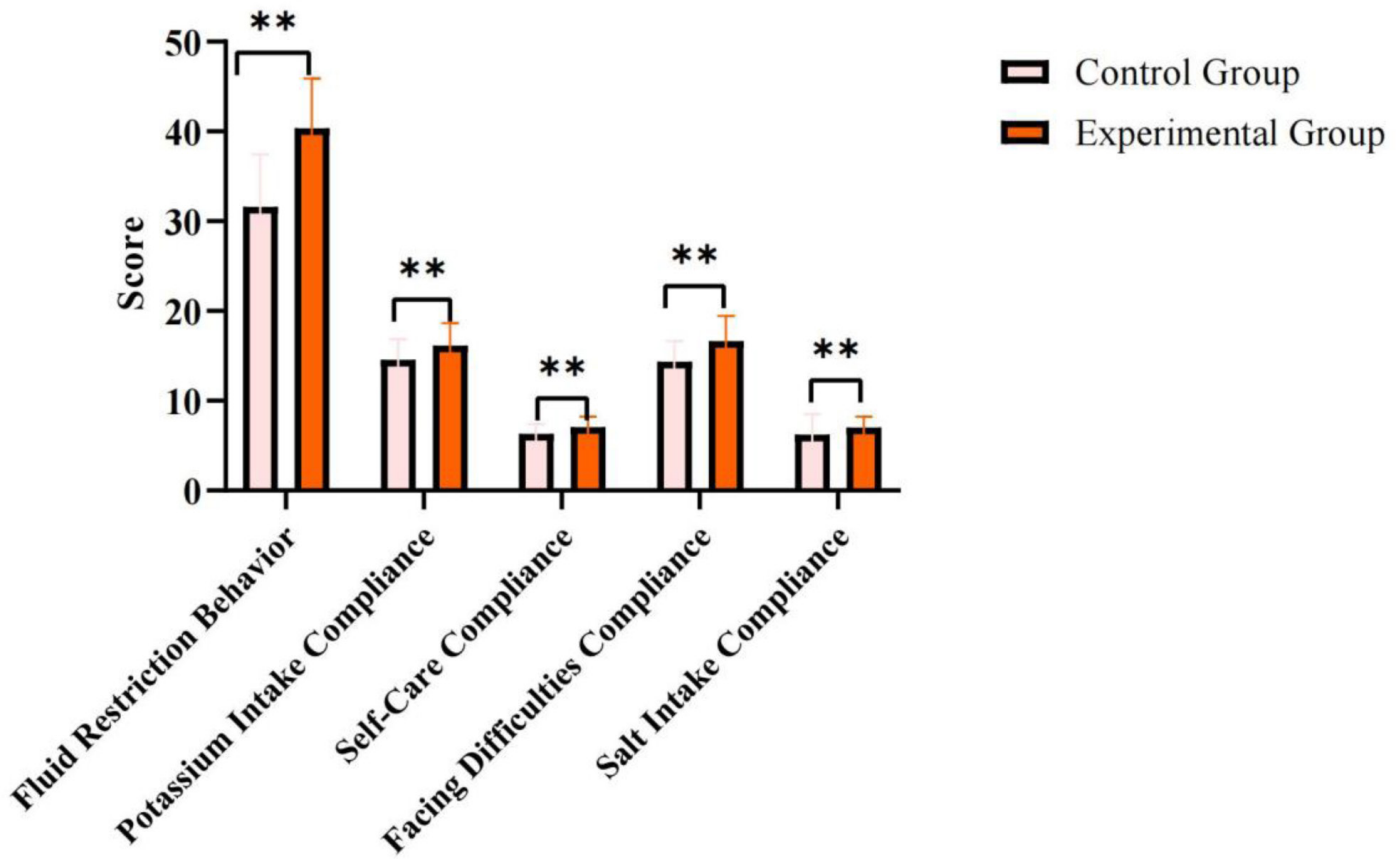
Comparison of dietary compliance between 2 groups of patients. Both the control group and the experimental group consist of 60 cases each. * Represents *P* < .05. ** Represents *P* < .01.

The results demonstrated that the experimental group exhibited significantly enhanced dietary compliance in various aspects compared to the control group, including fluid restriction, potassium intake control, self-care, disease management, and sodium intake (*P* < .05), thereby improving overall dietary management compliance, as shown in Figure 1.

### 3.3. Preventive treatment compliance results

**Figure 2.**
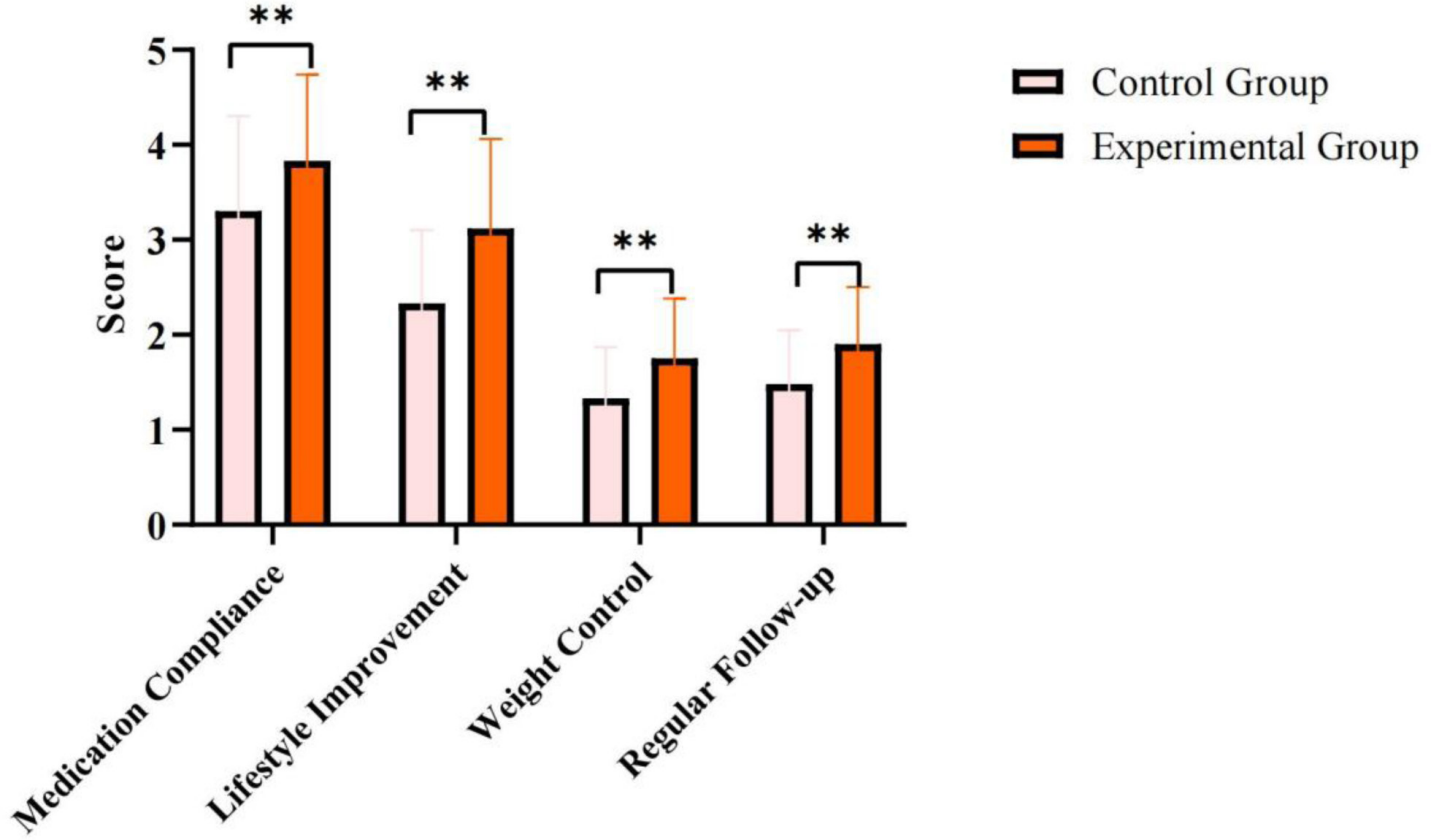
Comparison of preventive treatment compliance between the 2 groups. Both the control group and the experimental group consist of 60 cases each. * Represents *P* < .05. ** Represents *P* < .01.

Compared to the control group’s preventive-treatment compliance, the experimental group showed significantly higher scores in medication compliance, lifestyle improvement, weight control, and regular follow-up visits (*P* < .05), as detailed in Figure 2.

### 3.4. Negative emotions

**Figure 3.**
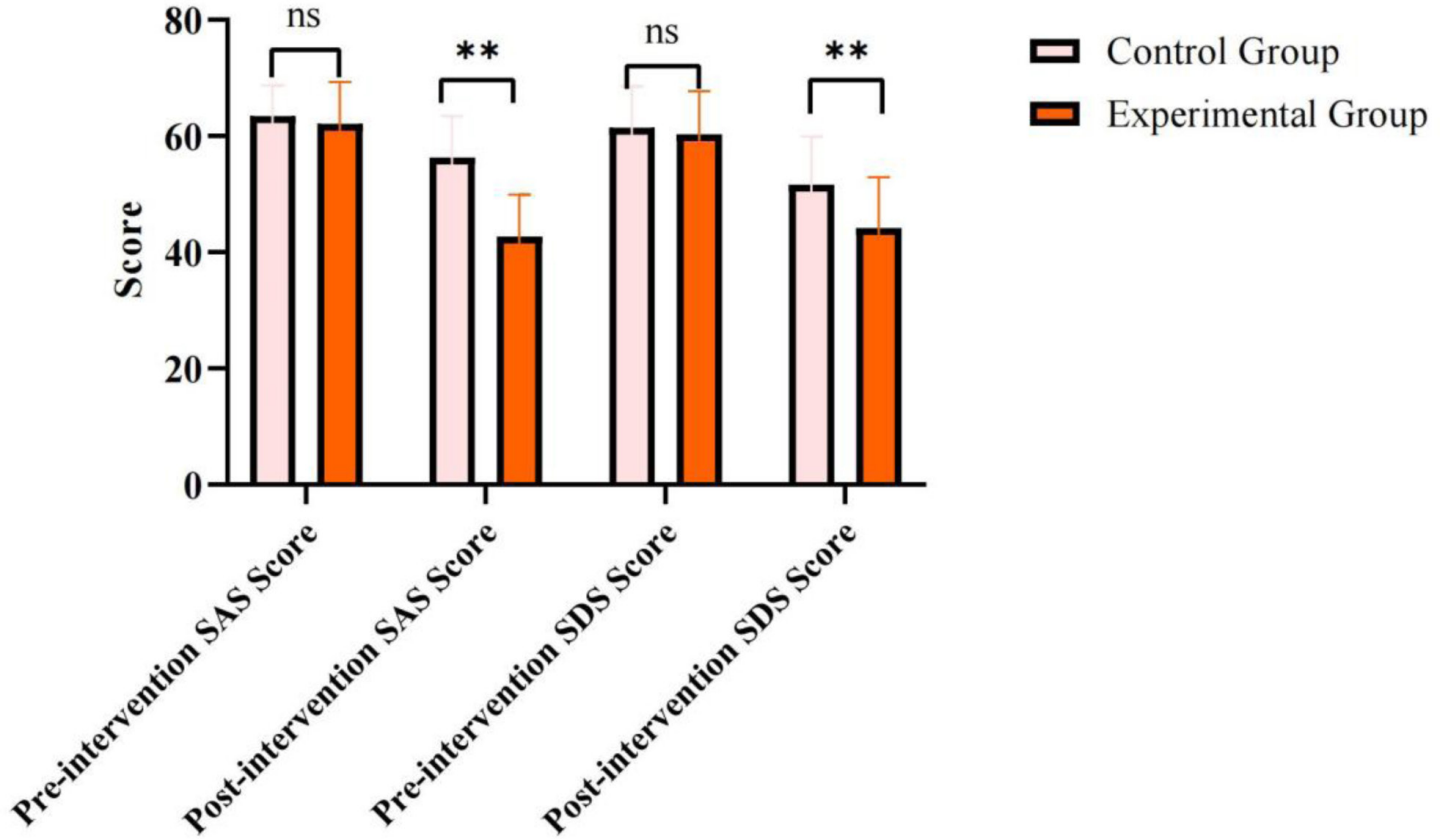
Comparison of SAS and SDS scores between the 2 groups. Both the control group and the experimental group consist of 60 cases each. ns indicates no difference between the 2 groups. * Represents *P* < .05. ** Represents *P* < .01. SAS = self-rating anxiety scale, SDS = self-rating depression scale.

There were no significant differences in the SAS and SDS scores between the control and experimental groups before the intervention (*P* > .05), indicating comparability between the 2 groups. After the intervention, the scores for both groups decreased, and the SAS and SDS scores of the experimental group were significantly lower than those of the control group (*P* < .05), as detailed in Figure 3.

### 3.5. Quality of life scores and nursing satisfaction

**Figure 4.**
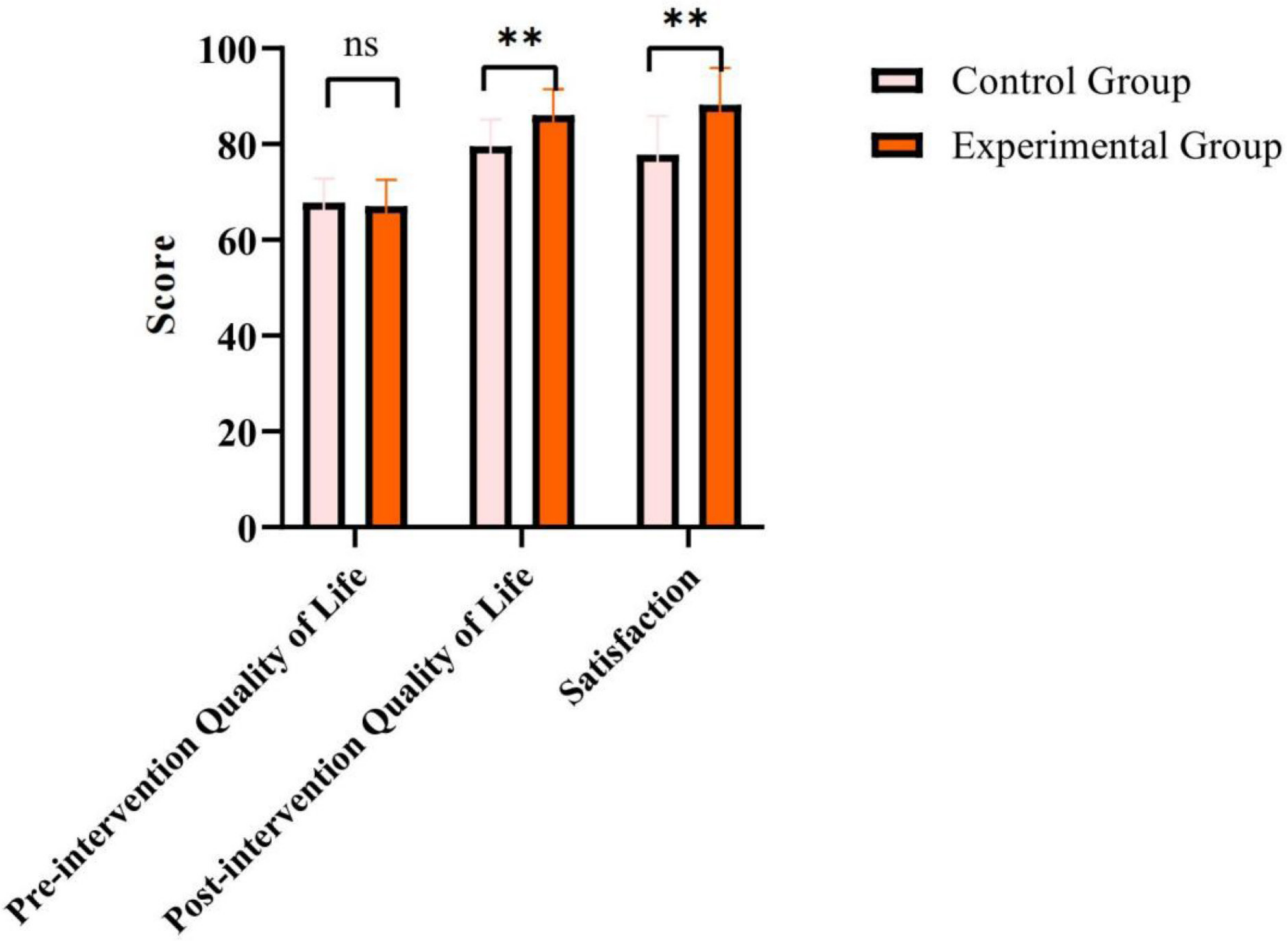
Comparison of quality of life scores and nursing satisfaction between the 2 groups. Both the control group and the experimental group consist of 60 cases each. ns indicates no difference between the 2 groups. * Represents *P* < .05. ** Represents *P* < .01.

While there were no significant differences in quality of life scores and nursing satisfaction between the groups pre-intervention (*P* > .05), the experimental group demonstrated significant improvements in both quality of life and nursing satisfaction post-intervention compared to the control group (*P* < .05), underscoring the positive impact of the intervention on these outcomes, as detailed in Figure 4.

## 4. Discussion

The prevalence of hyperkalemia, characterized by elevated potassium levels, varies among different populations. While the general populace typically exhibits a prevalence between 2% and 3%, outpatients in China have experienced an increased prevalence of 3.86%.^[15]^ Notably, individuals with CKD have seen a substantial rise in hyperkalemia rates, reaching 22.89%.^[16]^ The progression of CKD is intricately linked to the heightened prevalence of hyperkalemia, primarily attributed to the utilization of medications like renin-angiotensin-aldosterone system (RAAS) inhibitors. The presence of hyperkalemia in CKD patients is strongly associated with adverse outcomes, manifesting as a 90% elevated risk of cardiovascular events and a 65% increased mortality rate compared to CKD patients with normal potassium levels.^[17]^ In advanced CKD stages 4 to 5, the mortality rate escalates from 20.12% to 35.58% in the presence of hyperkalemia.^[18]^ Furthermore, hyperkalemia poses limitations on the use of RAAS inhibitors, heightening the risk of disease progression, hospitalizations, and medical expenses.

Recent research has underscored the significance of continuity of care in managing chronic ailments.^[19,20]^ This concept entails ensuring patients receive seamless and coordinated medical attention across healthcare services. For individuals grappling with chronic conditions such as hypertension, diabetes, chronic obstructive pulmonary disease, and CKD, continuity of care plays a pivotal role in enhancing treatment adherence, minimizing medical errors, and potentially reducing readmission rates.^[21]^

A recent study delved into the favorable impact of continuity of care on the compliance of patients dealing with CKD and hyperkalemia. The research revealed a notable correlation between enhanced compliance and improvements in quality of life and emotional well-being. Compliance, gauging the adherence to medical directives, is crucial for managing chronic illnesses. The study’s experimental group exhibited significantly better dietary compliance, encompassing aspects like fluid restriction, potassium intake control, self-care practices, coping with disease-related challenges, and sodium intake, compared to the control group. These findings align with existing literature highlighting the role of continuity of care in bolstering patients’ self-management capacities.^[22,23]^

The implementation of a continuity-of-care model offers tailored health education, consistent follow-ups, remote assistance, and fosters a collaborative network involving hospitals, communities, and families, thereby aiding patients in adhering to medical recommendations and enhancing their lifestyle. This approach not only boosts patient compliance but also markedly enhances their quality of life. Improved treatment adherence directly impacts patients’ quality of life, while personalized support and education alleviate negative emotions, indirectly bolstering compliance and quality of life. Patients’ high satisfaction with nursing services can serve as a catalyst for active engagement in self-management, further enhancing compliance. These interconnected outcome indicators form a closed-loop system reflecting the holistic health impact of continuity of care on individuals grappling with CKD and hyperkalemia.

Despite the merits of the continuity-of-care model, challenges may surface during its practical application. Resource allocation constraints can impede the delivery of personalized nursing services, while patient acceptance and engagement can influence the care’s effectiveness. To surmount these hurdles, a multifaceted strategy is recommended, encompassing optimized resource allocation, enhanced nursing service efficiency, heightened patient education, and the establishment of interdisciplinary teams comprising various healthcare professionals. Leveraging modern information technologies like mobile health applications and remote monitoring systems can bolster the accessibility and continuity-of-care services, especially in resource-constrained settings.

Although the present study suggests that a continuity-of-care nursing model is associated with improved adherence, psychological status, quality of life, and nursing satisfaction among patients with CKD complicated by hyperkalemia, several limitations should be acknowledged. First, this was a retrospective and non-randomized study; therefore, causal relationships cannot be firmly established. Patients were categorized according to documented nursing management and follow-up intensity rather than random allocation, which may have introduced selection bias. Second, although the 2 groups were comparable in several baseline demographic and clinical characteristics, unmeasured confounding factors may still have influenced the results. These factors may include socioeconomic status, educational level, family support, baseline self-management ability, baseline treatment adherence, health literacy, comorbidities, access to community medical resources, and motivation to participate in follow-up care. Patients with stronger family support or higher baseline adherence may have been more likely to benefit from, or actively engage with, the continuity-of-care model. Third, although propensity score matching or multivariable regression could reduce confounding bias, these methods were not applied in the present study because several key variables that may influence adherence and quality of life, such as socioeconomic status, family support, health literacy, and baseline self-management ability, were not consistently available in the retrospective records. Conducting propensity score matching using only a limited number of recorded variables may not adequately address confounding and may create a false impression of adjustment. Future studies should prospectively collect comprehensive baseline variables and use multivariable regression, propensity score matching, inverse probability weighting, or randomized controlled designs to better evaluate the independent effect of continuity of care.

## 5. Conclusion

The continuity-of-care model, by providing personalized and uninterrupted nursing services, significantly enhances treatment compliance and quality of life for individuals battling CKD and hyperkalemia, thereby holding immense value in ameliorating the prognosis and quality of life for such patients.

## Author contributions

**Conceptualization:** Yu Yan, Juan Liu, Guifang Liu.

**Data curation:** Yu Yan, Juan Liu, Guifang Liu.

**Formal analysis:** Yu Yan, Juan Liu, Guifang Liu.

**Funding acquisition:** Yu Yan, Guifang Liu.

**Investigation:** Yu Yan, Guifang Liu.

**Writing – original draft:** Yu Yan, Guifang Liu.

**Writing – review & editing:** Yu Yan, Guifang Liu.
